# Supplementary material for: Prenatal exposure to per- and polyfluoroalkyl substances (PFAS) and incidence of asthma and wheeze in childhood: A register-based cohort study in Ronneby, Sweden
Source: PLoS Med. 2026 Apr 9;23(4):e1004659. doi: 10.1371/journal.pmed.1004659 (PMC13065015; doi:10.1371/journal.pmed.1004659)
Supplement: S1 Table — (DOCX) [file pmed.1004659.s002.docx]

S1 Table: A summary of the different cohorts used in this study, including their date ranges and data sources.

| Cohort Name | Description | Date Range |  | Data Sources and Uses | | | | | | |
| --- | --- | --- | --- | --- | --- | --- | --- | --- | --- | --- |
|  |  |  | Total Population Register | Outcome | | | Exposure | | Covariates | |
|  |  |  |  | National Patient Register | National Prescribed Drug Register | Blekinge Healthcare Register | Historical water distribution records | Ronneby Biomarker Cohort | National Medical Birth Register | LISA* |
| Primary cohort  N_subj_ = 12,585 | Children born in Blekinge County between 2006-2013. Children were followed from birth until they either had a medical outcome, reached the outcome-specific maximum age, died, or were censored by emigration from Sweden or the end of the study. | January 1, 2006- December 31, 2022 | X | X | X |  | X |  | X | X |
| Exposure validation  N_subj_ = 209 | Women in the Ronneby Biomarker Cohort between ages 21-40 with known residential history the five years before sampling. | June 1, 2014- December 31, 2016 | X |  |  |  | X | X |  |  |
| Outcome validation  N_subj_ = 16,145 | Children born in Blekinge County between 2010-2021. Children were followed from birth until they either had a medical outcome, reached the outcome-specific maximum age, died, or were censored by emigration from Blekinge or the end of the study. | January 1, 2010-December 31, 2022 | X | X | X | X |  |  |  |  |

* Longitudinal Integrated Database for Health Insurance and Labor Market Studies
