# Supplementary material for: Prenatal exposure to per- and polyfluoroalkyl substances (PFAS) and incidence of asthma and wheeze in childhood: A register-based cohort study in Ronneby, Sweden
Source: PLoS Med. 2026 Apr 9;23(4):e1004659. doi: 10.1371/journal.pmed.1004659 (PMC13065015; doi:10.1371/journal.pmed.1004659)
Supplement: S2 Table — (DOCX) [file pmed.1004659.s003.docx]

S2 Table: Outcome definitions based on the National Patient Register (NPR), National Prescribed Drug Register (PDR), and primary care records from Region Blekinge (PC-Blekinge). Primary outcomes in the study were ascertained from the NPR and PDR records and validated against outcomes ascertained from the PC-Blekinge records.

| Outcome | Age Range | Data Source | Criteria |
| --- | --- | --- | --- |
| Asthma | 0-12 years | NPR | ≥1 ICD10 code for J45 (asthma). |
|  |  | PDR | ≥2 prescriptions of R03BA (inhalations of glucocorticoids), R03DC (leukotriene receptor antagonists), or R03AK (fixed combinations of inhalations of β2-agonists and corticosteroids) with ≥ 2-week gap between distributions (age 0-4.5) or regardless of timing (age >4.5). |
|  |  |  | ≥3 prescriptions of R03AC (inhalations of selective β2-adrenoreceptor agonists). |
|  |  | PC-Blekinge | ≥1 ICD10 code for J45 (asthma). |
| Asthma (3+) | 0-12 years | NPR and PDR | Subject meets the criteria for asthma at least once at age 36 months or older (either in the NPR and/or PDR). Incidence date is still the first occurrence of an asthma diagnosis or dispensation. |
|  |  | PC-Blekinge | ≥1 ICD10 code for J45 (asthma) at age 36 months or older. |
| Wheeze | 0-36 months | NPR | ≥1 ICD10 code for J45 (asthma) or J20-J22 (other acute infections in the lower airways). |
|  |  | PDR | ≥1 prescription of R03BA (glucocorticoids), R03DC (leukotriene receptor antagonists), R03AK (fixed combinations of β2-agonists and corticosteroids) or R03AC (inhalations of selective β2-adrenoreceptor agonists) |
|  |  | PC-Blekinge | ≥1 ICD10 code for J45 (asthma) or J20-J22 (other acute infections in the lower airways). |
