# Supplementary material for: Prenatal exposure to per- and polyfluoroalkyl substances (PFAS) and incidence of asthma and wheeze in childhood: A register-based cohort study in Ronneby, Sweden
Source: PLoS Med. 2026 Apr 9;23(4):e1004659. doi: 10.1371/journal.pmed.1004659 (PMC13065015; doi:10.1371/journal.pmed.1004659)
Supplement: S3 Table — (DOCX) [file pmed.1004659.s004.docx]

S3 Table: Baseline cohort characteristics for children with and without complete covariate data, displayed as N (%) or median [interquartile range].

| Variable | | Overall | Complete covariate information | Incomplete covariate information |
| --- | --- | --- | --- | --- |
| N |  | 12585 | 11488 | 1097 |
| Maternal smoking in early pregnancy | Non-smoker | 10909 (86.7) | 10587 (92.2) | 322 (29.4) |
|  | Smoker | 957 (7.6) | 901 (7.8) | 56 (5.1) |
|  | Missing | 719 (5.7) | 0 (0.0) | 719 (65.5) |
| Parity | Primiparous | 5232 (41.6) | 4814 (41.9) | 418 (38.1) |
|  | Multiparous | 7206 (57.3) | 6674 (58.1) | 532 (48.5) |
|  | Missing | 147 (1.2) | 0 (0.0) | 147 (13.4) |
| Sex | Male | 6517 (51.8) | 5949 (51.8) | 568 (51.8) |
|  | Female | 6068 (48.2) | 5539 (48.2) | 529 (48.2) |
| Maternal education | Primary and lower secondary | 2507 (19.9) | 2292 (20.0) | 215 (19.6) |
|  | Upper secondary | 3884 (30.9) | 3630 (31.6) | 254 (23.2) |
|  | Post secondary | 5926 (47.1) | 5566 (48.5) | 360 (32.8) |
|  | Missing | 268 (2.1) | 0 (0.0) | 268 (24.4) |
| Maternal age at delivery |  | 30.0 [26.3, 33.7] | 30.0 [26.4, 33.7] | 29.4 [24.9, 33.6] |
| At least one parent born abroad | False | 9845 (78.2) | 9265 (80.6) | 580 (52.9) |
|  | True | 2740 (21.8) | 2223 (19.4) | 517 (47.1) |
| Family disposable income  (Swedish krona x 10^5^) |  | 3.7 [2.8, 4.5] | 3.8 [3.0, 4.5] | 2.6 [1.4, 4.1] |
|  | Missing | 9 (0) | 0 | 9 (1.0) |
| Parental asthma | False | 10277 (81.7) | 9525 (82.9) | 752 (68.6) |
|  | True | 2089 (16.6) | 1963 (17.1) | 126 (11.5) |
|  | Missing | 219 (1.7) | 0 (0.0) | 219 (20.0) |
| Prenatal exposure | Background | 10203 (81.1) | 9224 (80.3) | 979 (89.2) |
|  | Intermediate | 1668 (13.3) | 1591 (13.8) | 77 (7.0) |
|  | High | 512 (4.1) | 479 (4.2) | 33 (3.0) |
|  | Very High | 202 (1.6) | 194 (1.7) | 8 (0.7) |
