# Supplementary material for: Prenatal exposure to per- and polyfluoroalkyl substances (PFAS) and incidence of asthma and wheeze in childhood: A register-based cohort study in Ronneby, Sweden
Source: PLoS Med. 2026 Apr 9;23(4):e1004659. doi: 10.1371/journal.pmed.1004659 (PMC13065015; doi:10.1371/journal.pmed.1004659)
Supplement: S4 Table — (DOCX) [file pmed.1004659.s005.docx]

S4 Table: Outcome algorithm performance compared to outcomes ascertained from Blekinge primary care records in a cohort of children born and residing in Blekinge, 2010-2021 (N = 16,145).

| Outcome | Sensitivity | Specificity | Positive Predictive Value | Negative Predictive Value |
| --- | --- | --- | --- | --- |
| Asthma | 0.83 | 0.96 | 0.75 | 0.98 |
| Asthma (3+) | 0.86 | 0.97 | 0.63 | 0.99 |
| Wheeze | 0.76 | 0.93 | 0.70 | 0.95 |
