# Supplementary material for: Prenatal exposure to per- and polyfluoroalkyl substances (PFAS) and incidence of asthma and wheeze in childhood: A register-based cohort study in Ronneby, Sweden
Source: PLoS Med. 2026 Apr 9;23(4):e1004659. doi: 10.1371/journal.pmed.1004659 (PMC13065015; doi:10.1371/journal.pmed.1004659)
Supplement: S5 Table — (DOCX) [file pmed.1004659.s006.docx]

S5 Table: Pooled hazard ratios calculated from 20 multiple-imputed datasets.

| Prenatal Exposure Group | Events | Person-Years | Hazard Ratio (95% Confidence Interval) | |
| --- | --- | --- | --- | --- |
|  |  |  | Simple model^1^ | Adjusted model^2^ |
| **Wheeze** |  |  |  |  |
| Background | 1814 | 27318 | - | - |
| Intermediate | 112 | 1341 | 1.26 (1.02, 1.56) | 1.11 (0.89, 1.37) |
| High | 295 | 4474 | 1 (0.88, 1.14) | 1 (0.87, 1.13) |
| Very High | 39 | 530 | 1.11 (0.79, 1.55) | 0.98 (0.7, 1.37) |
| **Asthma** |  |  |  |  |
| Background | 1759 | 105592 | - | - |
| Intermediate | 98 | 5218 | 1.13 (0.9, 1.42) | 0.99 (0.78, 1.24) |
| High | 269 | 17473 | 0.94 (0.82, 1.07) | 0.93 (0.82, 1.07) |
| Very High | 51 | 2000 | 1.52 (1.14, 2.02) | 1.40 (1.05, 1.86) |
| **Asthma (3+)** |  |  |  |  |
| Background | 1294 | 110544 | - | - |
| Intermediate | 69 | 5530 | 1.07 (0.83, 1.39) | 0.96 (0.74, 1.25) |
| High | 191 | 18311 | 0.90 (0.77, 1.06) | 0.90 (0.77, 1.05) |
| Very High | 40 | 2125 | 1.60 (1.15, 2.21) | 1.51 (1.09, 2.09) |

1. Baseline hazard is stratified by child sex.
2. Baseline hazard is stratified by child sex and maternal parity (primiparous or multiparous). The model is also adjusted for the following covariates: maternal smoking status in early pregnancy (smoker or non-smoker); maternal education (primary and lower secondary, upper secondary, and post-secondary); at least one foreign-born parent (yes or no); family disposable income (quantiles); maternal age at delivery (quantiles), and maternal asthma (yes or no).
