# Supplementary material for: Prenatal exposure to per- and polyfluoroalkyl substances (PFAS) and incidence of asthma and wheeze in childhood: A register-based cohort study in Ronneby, Sweden
Source: PLoS Med. 2026 Apr 9;23(4):e1004659. doi: 10.1371/journal.pmed.1004659 (PMC13065015; doi:10.1371/journal.pmed.1004659)
Supplement: S6 Table — (DOCX) [file pmed.1004659.s007.docx]

S6 Table: Hazard ratios for each outcome stratified by sex. Models include a baseline hazard stratified by maternal parity (primiparous or multiparous) and are adjusted for the following covariates: maternal smoking status in early pregnancy (smoker or non-smoker); maternal education (primary and lower secondary, upper secondary, and post-secondary); at least one foreign-born parent (yes or no); family disposable income (quantiles); maternal age at delivery (quantiles), and parental asthma (yes or no).

| Prenatal Exposure Group | Events | Person-Years | Adjusted Hazard Ratio (95% Confidence Interval) |
| --- | --- | --- | --- |
| **Wheeze – Male** |  |  |  |
| Background | 1008 (21%) | 12579 | - |
| Intermediate | 159 (20%) | 2085 | 0.94 (0.79, 1.12) |
| High | 58 (24%) | 622 | 1.02 (0.77, 1.34) |
| Very High | 30 (28%) | 260 | 1.23 (0.84, 1.79) |
| **Wheeze – Female** |  |  |  |
| Background | 653 (15%) | 12099 | - |
| Intermediate | 125 (16%) | 2184 | 1.08 (0.89, 1.32) |
| High | 45 (19%) | 633 | 1.18 (0.86, 1.62) |
| Very High | 7 (8%) | 249 | 0.50 (0.24, 1.04) |
| **Asthma – Male** |  |  |  |
| Background | 969 (20%) | 48651 | - |
| Intermediate | 151 (19%) | 8104 | 0.93 (0.78, 1.11) |
| High | 50 (21%) | 2417 | 0.91 (0.67, 1.23) |
| Very High | 37 (35%) | 951 | 1.69 (1.21, 2.37) |
| **Asthma – Female** |  |  |  |
| Background | 627 (14%) | 47309 | - |
| Intermediate | 110 (14%) | 8624 | 0.98 (0.8, 1.21) |
| High | 39 (16%) | 2507 | 1.04 (0.74, 1.46) |
| Very High | 13 (15%) | 968 | 0.99 (0.59, 1.67) |
| **Asthma (3+) – Male** |  |  |  |
| Background | 707 (15%) | 51467 | - |
| Intermediate | 109 (14%) | 8549 | 0.92 (0.75, 1.13) |
| High | 34 (14%) | 2587 | 0.85 (0.59, 1.23) |
| Very High | 29 (27%) | 1041 | 1.81 (1.23, 2.66) |
| **Asthma (3+) – Female** |  |  |  |
| Background | 463 (10%) | 49051 | - |
| Intermediate | 77 (10%) | 8982 | 0.93 (0.73, 1.18) |
| High | 29 (12%) | 2620 | 1.06 (0.73, 1.55) |
| Very High | 11 (13%) | 992 | 1.20 (0.68, 2.11) |
