# Supplementary material for: Prenatal exposure to per- and polyfluoroalkyl substances (PFAS) and incidence of asthma and wheeze in childhood: A register-based cohort study in Ronneby, Sweden
Source: PLoS Med. 2026 Apr 9;23(4):e1004659. doi: 10.1371/journal.pmed.1004659 (PMC13065015; doi:10.1371/journal.pmed.1004659)
Supplement: S1 Fig — (DOCX) [file pmed.1004659.s008.docx]

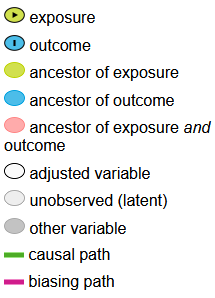

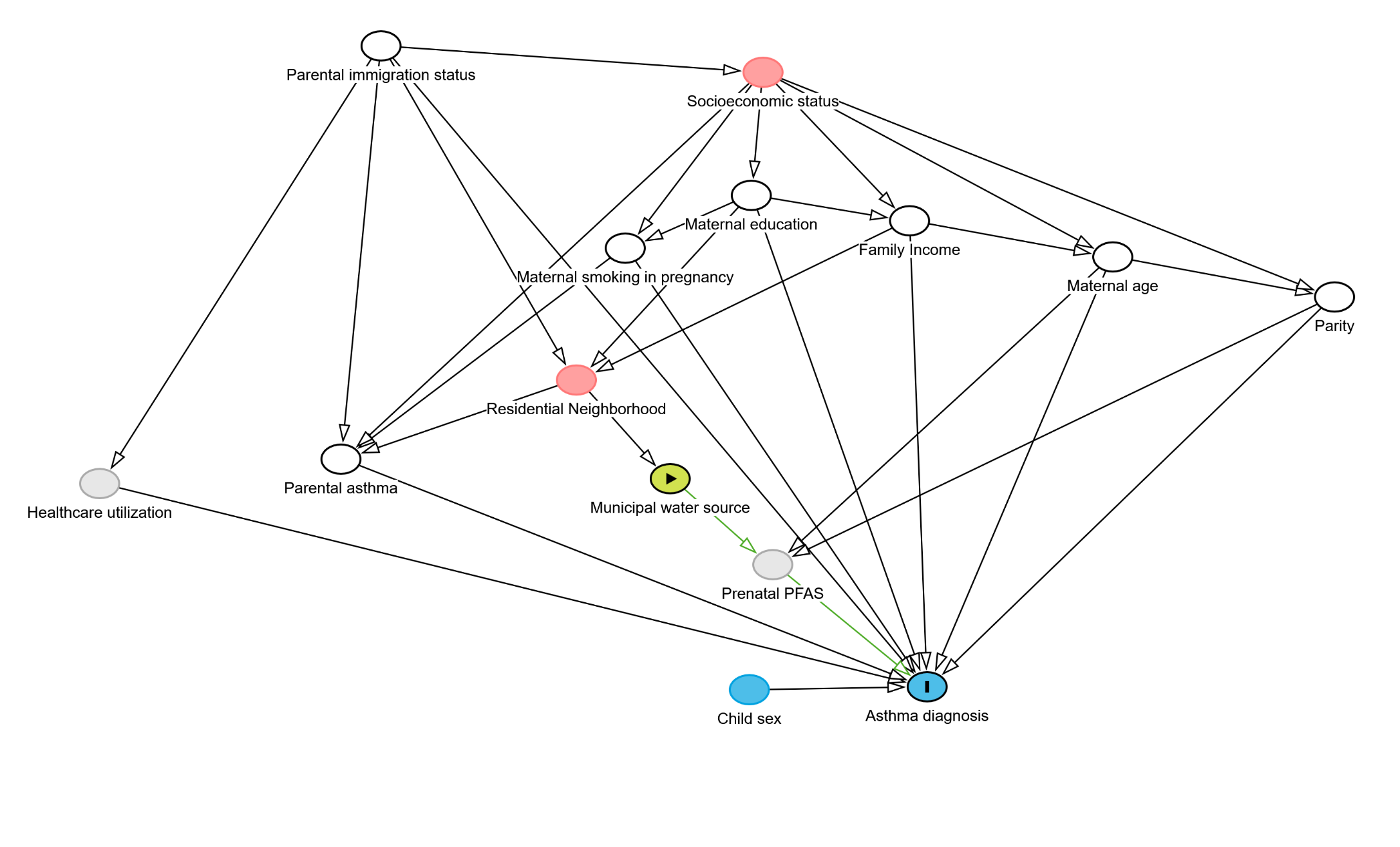


S1 Figure: Directed acyclic graph (DAG) for the association between PFAS exposure via the municipal water source and diagnosis of childhood asthma. Variables in grey are unmeasured. Variables in white are the minimally sufficient adjustment set for the total effect, and were adjusted for in our primary models.
