# Supplementary material for: Prenatal exposure to per- and polyfluoroalkyl substances (PFAS) and incidence of asthma and wheeze in childhood: A register-based cohort study in Ronneby, Sweden
Source: PLoS Med. 2026 Apr 9;23(4):e1004659. doi: 10.1371/journal.pmed.1004659 (PMC13065015; doi:10.1371/journal.pmed.1004659)
Supplement: S2 Fig — (DOCX) [file pmed.1004659.s009.docx]

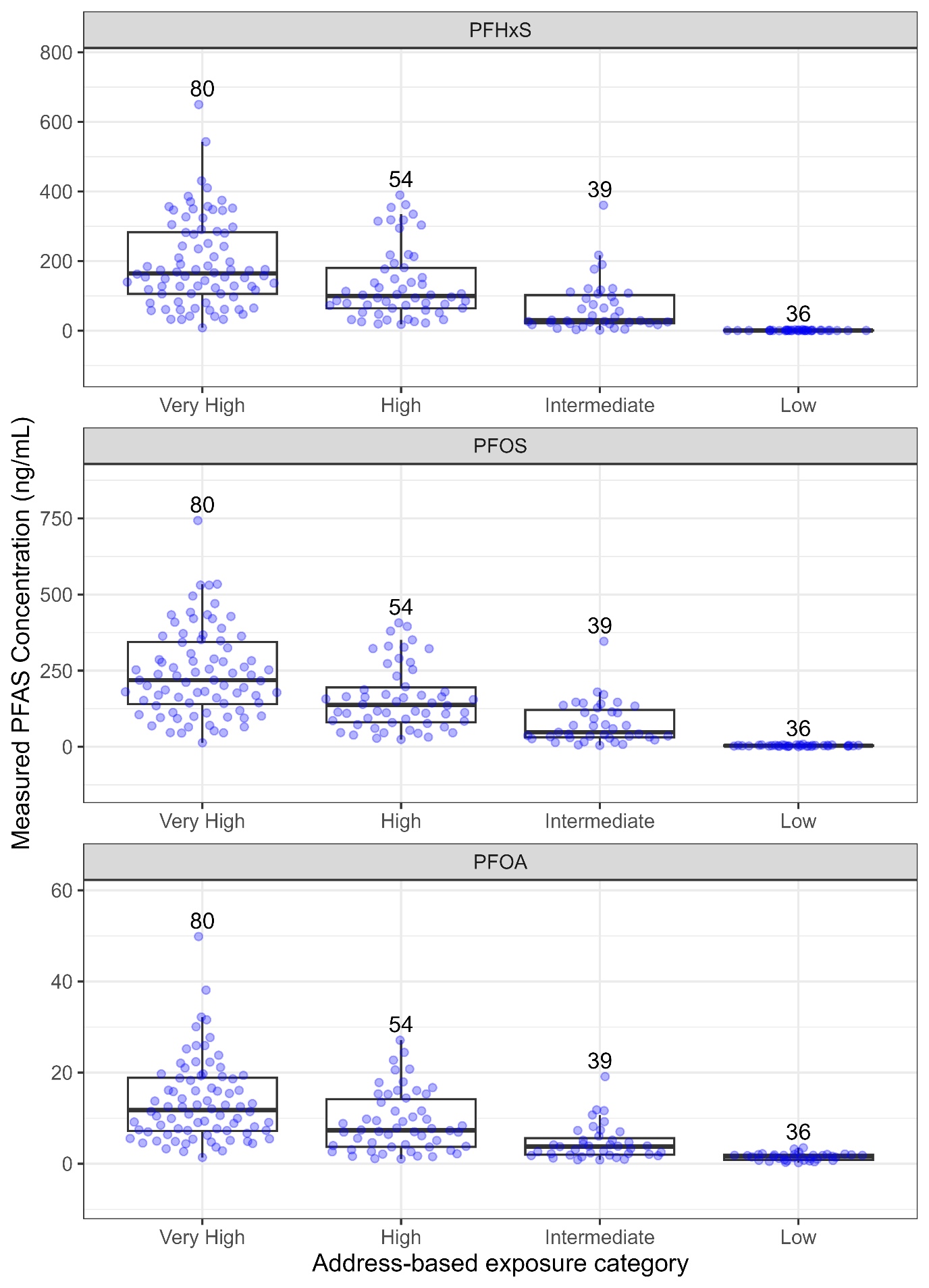


S2 Figure: PFAS concentrations (ng/mL) in a subset of the Ronneby Biomarker Cohort (female subjects aged 21-40 years, N = 209), stratified by categorical exposure groups. The box plots show the median (center line), interquartile range (box), and whiskers extending to 1.5 times the interquartile range; individual observations are shown in blue.
