# Supplementary material for: Prenatal exposure to per- and polyfluoroalkyl substances (PFAS) and incidence of asthma and wheeze in childhood: A register-based cohort study in Ronneby, Sweden
Source: PLoS Med. 2026 Apr 9;23(4):e1004659. doi: 10.1371/journal.pmed.1004659 (PMC13065015; doi:10.1371/journal.pmed.1004659)
Supplement: S4 Fig — (DOCX) [file pmed.1004659.s011.docx]

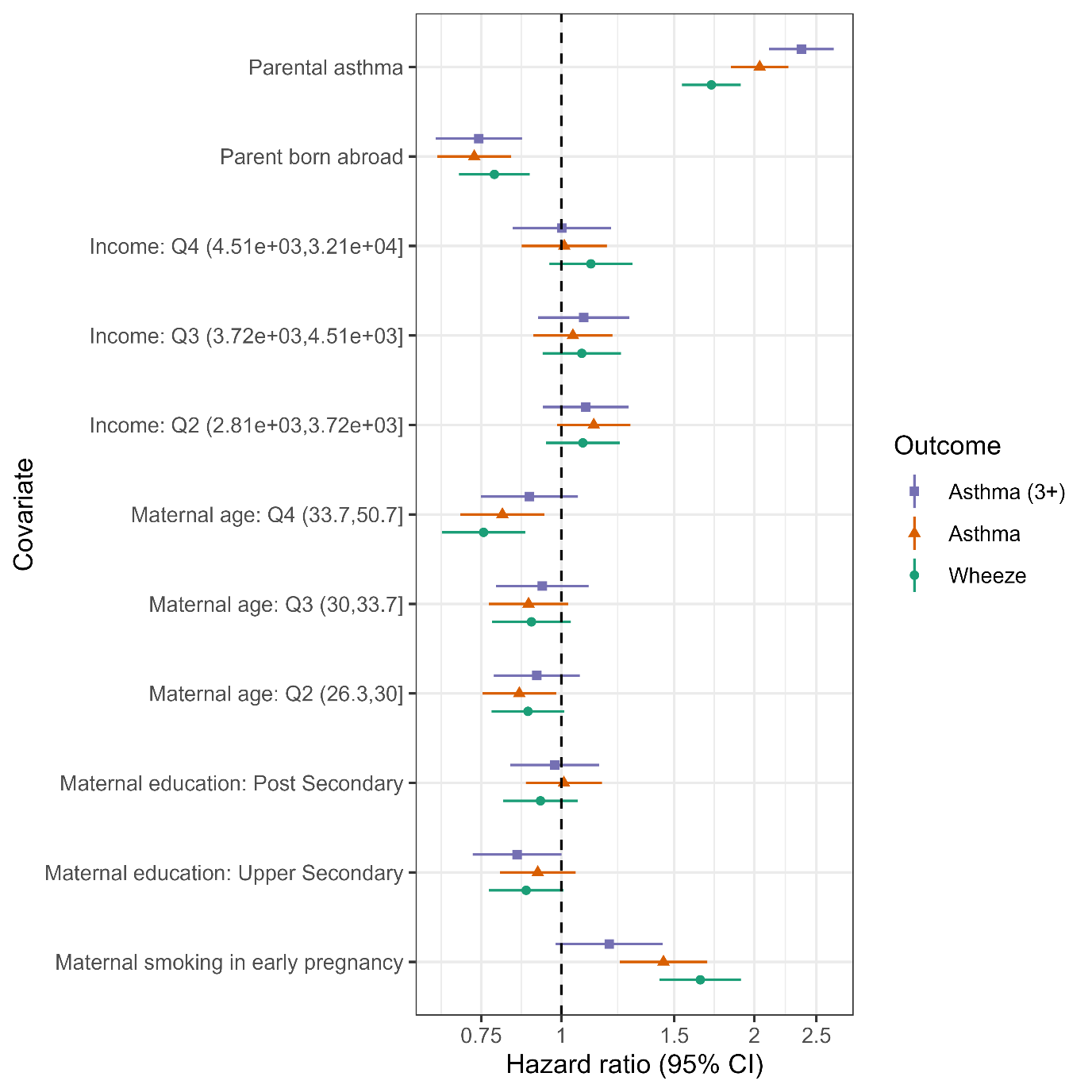


S4 Figure: Hazard ratios for covariates from the primary adjusted models, which also included a baseline hazard stratified by sex and by maternal parity.

Q2 = second quartile; Q3 = third quartile; Q4 = fourth quartile.
