# Supplementary material for: Prenatal exposure to per- and polyfluoroalkyl substances (PFAS) and incidence of asthma and wheeze in childhood: A register-based cohort study in Ronneby, Sweden
Source: PLoS Med. 2026 Apr 9;23(4):e1004659. doi: 10.1371/journal.pmed.1004659 (PMC13065015; doi:10.1371/journal.pmed.1004659)
Supplement: S5 Fig — (DOCX) [file pmed.1004659.s012.docx]

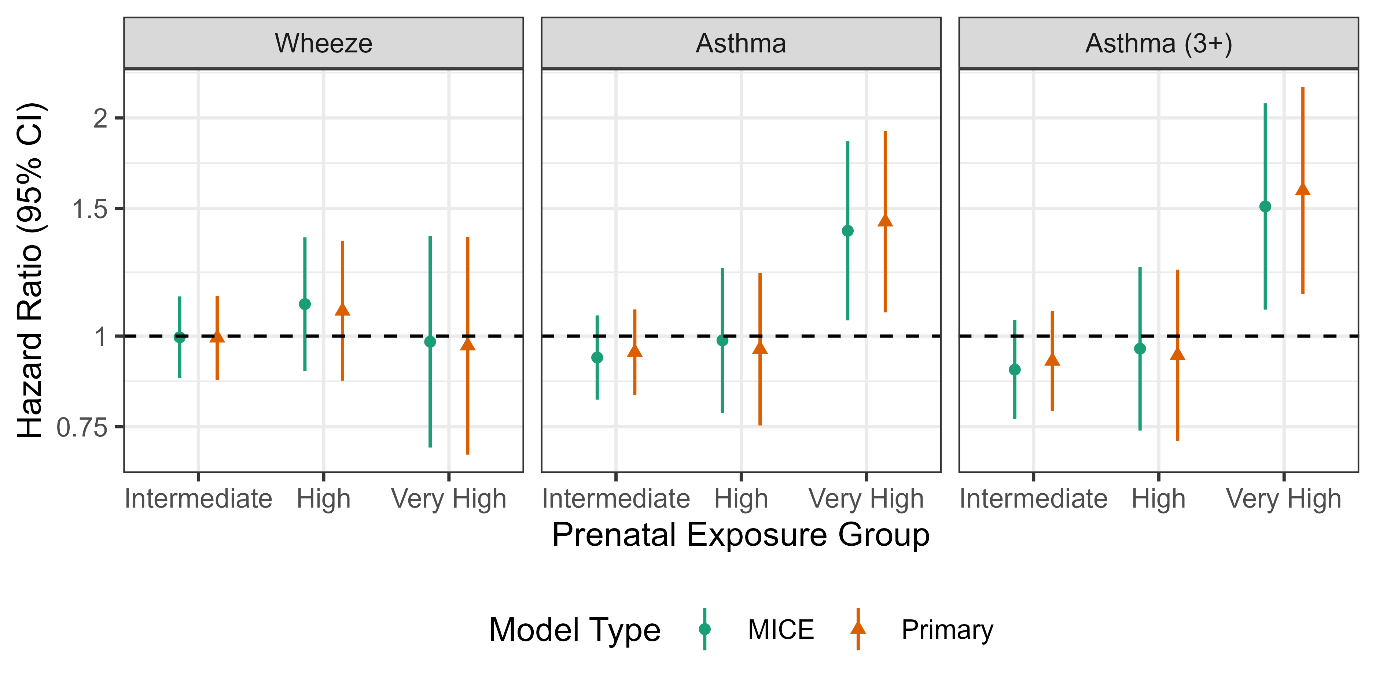


S5 Figure: Hazard ratios and 95% confidence intervals (“CI”) from the primary complete case analysis (“Primary”) compared to pooled ratios calculated using the multiple-imputed datasets (“MICE”). All models included a baseline hazard stratified by child sex and maternal parity (primiparous or multiparous) and were adjusted for the following covariates: maternal smoking in early pregnancy (smoker or non-smoker); maternal education (primary and lower secondary, upper secondary, and post-secondary); at least one foreign-born parent (yes or no); family disposable income (quantiles); maternal age at delivery (quantiles), and parental asthma (yes or no).
