# Supplementary material for: Prenatal exposure to per- and polyfluoroalkyl substances (PFAS) and incidence of asthma and wheeze in childhood: A register-based cohort study in Ronneby, Sweden
Source: PLoS Med. 2026 Apr 9;23(4):e1004659. doi: 10.1371/journal.pmed.1004659 (PMC13065015; doi:10.1371/journal.pmed.1004659)
Supplement: S6 Fig — (DOCX) [file pmed.1004659.s013.docx]

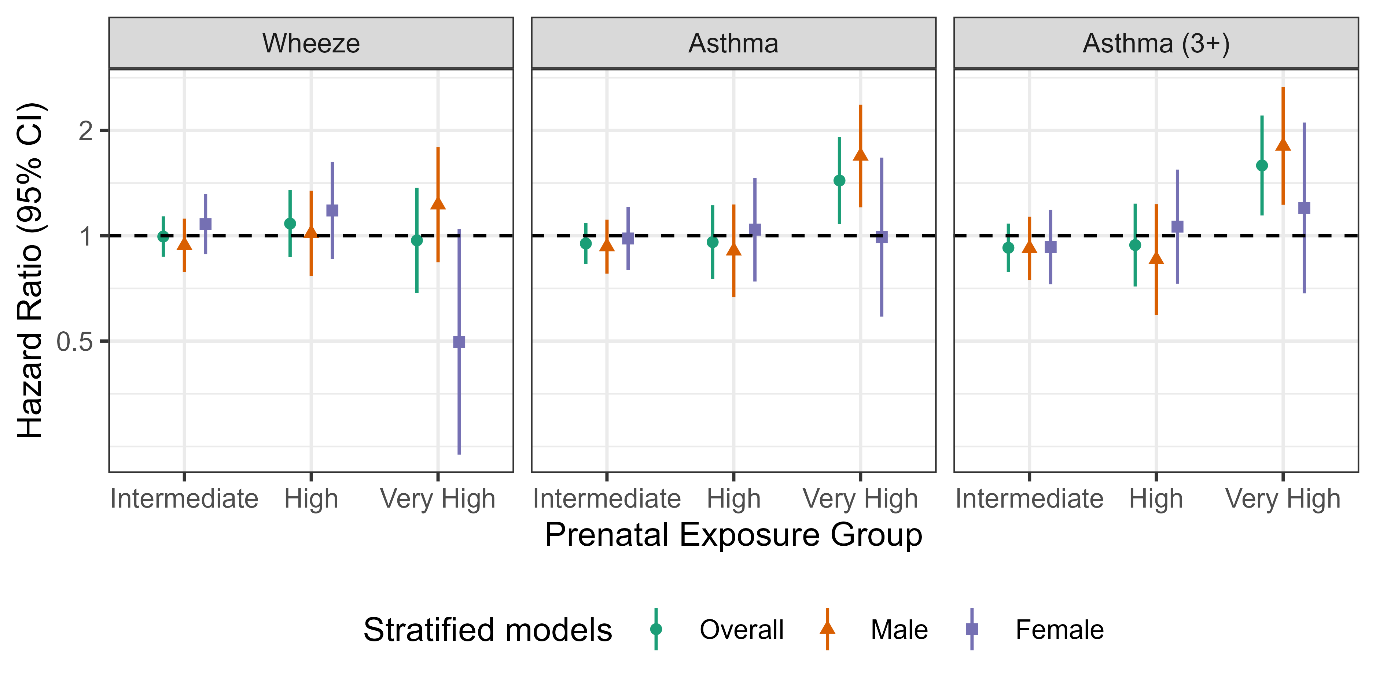


S6 Figure: Hazard ratios and 95% confidence intervals (“CI”) from the primary models stratified by sex. Sex-specific and combined models include a baseline hazard stratified by maternal parity (primiparous or multiparous) and are adjusted for the following covariates: maternal smoking status in early pregnancy (smoker or non-smoker); maternal education (primary and lower secondary, upper secondary, and post-secondary); at least one foreign-born parent (yes or no); family disposable income (quantiles); maternal age at delivery (quantiles), and parental asthma (yes or no). The combined model also includes a baseline hazard stratified by sex.
