# Supplementary material for: Prenatal exposure to per- and polyfluoroalkyl substances (PFAS) and incidence of asthma and wheeze in childhood: A register-based cohort study in Ronneby, Sweden
Source: PLoS Med. 2026 Apr 9;23(4):e1004659. doi: 10.1371/journal.pmed.1004659 (PMC13065015; doi:10.1371/journal.pmed.1004659)
Supplement: S7 Fig — (DOCX) [file pmed.1004659.s014.docx]

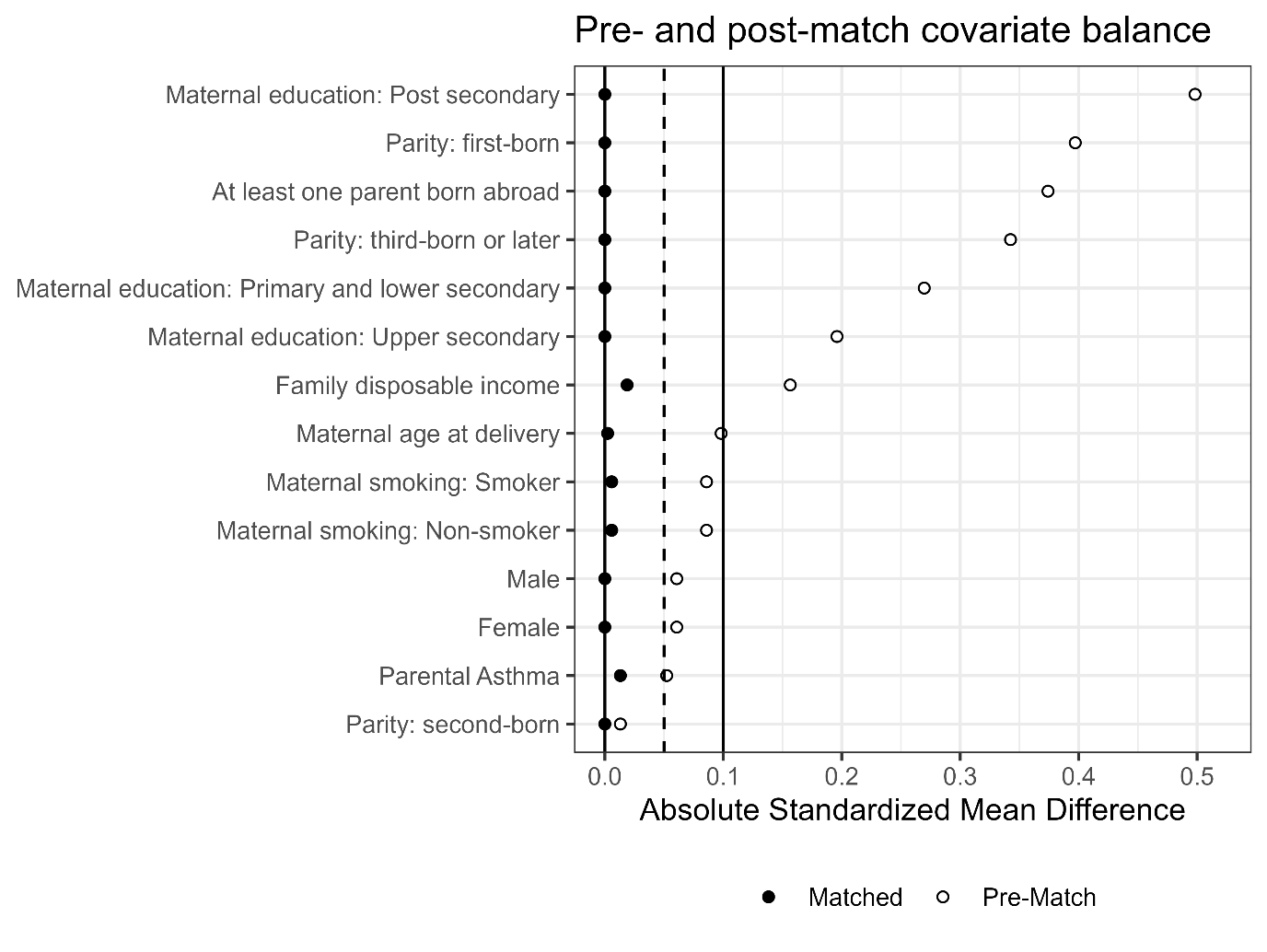


S7 Figure: Love plot showing the absolute standardized differences in covariate means between very high-exposed and background-exposed children before matching (“Pre-Match”) and after matching (“Matched") using robust rank-based Mahalanobis distance.
