# Supplementary material for: Prenatal exposure to per- and polyfluoroalkyl substances (PFAS) and incidence of asthma and wheeze in childhood: A register-based cohort study in Ronneby, Sweden
Source: PLoS Med. 2026 Apr 9;23(4):e1004659. doi: 10.1371/journal.pmed.1004659 (PMC13065015; doi:10.1371/journal.pmed.1004659)
Supplement: S8 Fig — (DOCX) [file pmed.1004659.s015.docx]

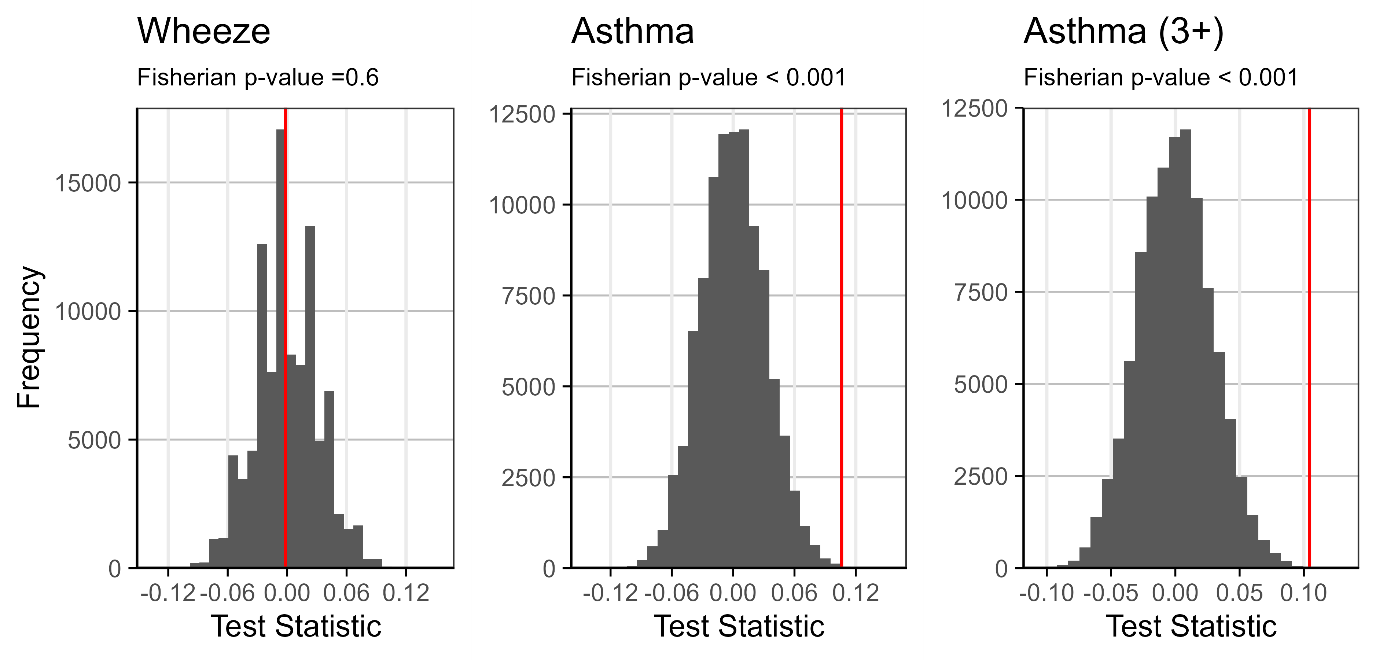


S8 Figure: Null randomization distributions of the test statistic (estimated difference in outcome-specific cumulative incidence by the end of the study period) used for the approximation of the Fisher exact p-value (N*_permutations_* = 100,000). The vertical red line indicates the observed difference in cumulative incidence.
