# Supplementary material for: Prenatal exposure to per- and polyfluoroalkyl substances (PFAS) and incidence of asthma and wheeze in childhood: A register-based cohort study in Ronneby, Sweden
Source: PLoS Med. 2026 Apr 9;23(4):e1004659. doi: 10.1371/journal.pmed.1004659 (PMC13065015; doi:10.1371/journal.pmed.1004659)
Supplement: Attachment — Submitted filename: Reviewer-and-Editorial-Responses-2-2-1.docx [file pmed.1004659.s018.docx]

Response to Editor Comments: PMEDICINE-D-25-01709-R1

Dear Editors and reviewers:

Thank you for the thoughtful comments provided on our manuscript, “Prenatal exposure to perfluoroalkyl substances (PFAS) and incidence of asthma and wheeze in childhood: A cohort study in Ronneby, Sweden” (PMEDICINE-D-25-01709-R1). We appreciate the opportunity to improve the manuscript based on your suggestions. All line numbers in our responses refer to the track-changes version.

# Reviewer 1

## Main Queries

1. It is unclear why the main analysis is not that using the imputed dataset and then accounting for clustering using this dataset. This is the more robust analysis and given there are siblings within the cohort the independence assumption of the model is violated in their current complete case primary analysis and should be avoided.

Thank you for this important point. We agree that accounting for within-family clustering improves the robustness of the analysis. We have therefore revised our primary Cox proportional hazards models to use cluster-robust variance estimators to account for correlation within siblings, and updated all corresponding statistical tests. See, for example:

“We then applied Cox proportional hazard models to estimate the association between prenatal PFAS exposure and disease incidence, using robust variance estimators to account for potential residual correlation within siblings.” (Line 234-236)

“We formally assessed the proportional hazards assumption for our categorical exposure variable by jointly testing the time-by-exposure interaction terms using robust Wald tests, which account for the cluster-robust covariance estimates from the fitted Cox models.” (Line 240-243)

Regarding imputation, our primary analysis now uses the cluster-robust complete-case models, and we use a sensitivity analysis to confirm that primary results were highly consistent with results from the multiply-imputed datasets. Given the low proportion of missing data (<8%) and the stability of estimates across imputed datasets, we retained the complete-case results as our main models for clarity of presentation.

2. How was the independence assumption handled in the unadjusted Kaplan-Meier analysis?

We appreciate this insightful comment. The reviewer is correct that standard Kaplan–Meier estimation and log-rank tests assume independent outcomes. While methods that account for clustering exist, they are rarely applied when Kaplan–Meier curves are used primarily for descriptive visualization. To clarify this, we have removed the log-rank tests from the KM analysis and now explicitly state that all formal inference relies on Cox proportional hazards models with cluster-robust covariance estimates.

“We used the Kaplan-Meier method to visualize the cumulative incidence of each outcome by prenatal PFAS exposure group, without adjustment for confounding variables. For formal inference, we applied Cox proportional hazard models to estimate the association between prenatal PFAS exposure and disease incidence, using robust variance estimators to account for potential residual correlation within siblings [49,50].” (Line 231-236)

3. Was there an a-priori statistical analysis plan? If so, this should be included in the supplemental files.

No, this study did not include an a-priori statistical analysis plan.

4. Further details of the causal analysis are needed, was there a trial target framework established a-priori or consideration of casual assumptions. Were the imputed data used for this analysis?

Thank you for this question. The analysis was conducted within subjects with complete covariate data. We believe this to be adequate, as over 96% of high-exposed subjects had complete data and the final matched dataset showed good balance across all covariates. We have clarified this in the methods section:

“Matching… was restricted to children with complete covariate data.” (Line 283-288).

The causal analysis utilizes a Rubin Causal Model framework and follows the recommendations of Bind and Rubin to embed an observational study in a hypothetical randomized experiment (Bind and Rubin, 2019). While similar in many respects to target trial emulation, we did not construct an a-priori TTE framework (although we appreciate the suggestion, and will consider this for future studies). We have rewritten the methods section of our study to be more explicit about the causal study design and its assumptions:

“As a secondary analysis, we used a Rubin Causal Model potential outcomes framework to assess a possible causal effect of very high versus background prenatal PFAS exposure on childhood wheeze and asthma. We constructed a hypothetical randomized experiment by matching each child in the very high exposure group to three controls from the background group using 3:1 nearest neighbor matching with robust rank-based Mahalanobis distance [57–60]. (Line 272-283)

“Causal interpretation of this secondary analysis relies on the assumptions outlined in Imbens & Rubin (2015): the stable unit treatment assignment value assumption (SUTVA), where there is no interference between units and the treatment is well-defined (i.e., consistency); probabilistic assignment mechanism, where every unit has a non-zero probability of each treatment; and unconfoundedness, where exposure is independent of potential outcomes after matching on background covariates [62].” (Line 302-308)

5. In the causal matched analysis, were the models adjusted for post matching? This is recommended to account for residual imbalance and doubly robust estimation.

Thank you for this helpful comment. As described in the Methods section, our causal analysis was based on hypothesis testing of a test statistic—cumulative incidence by maximum age—estimated from Kaplan–Meier survival models. We did not include additional covariate adjustment post-matching. While post-matching adjustment can be applied to account for residual imbalance or to achieve doubly robust estimation, our matched cohort showed excellent covariate balance across all potential confounders (S14 Figure). Given this, additional adjustment was unlikely to materially change the results, and we therefore relied on the matched design as our primary causal approach.

6. Line 327 – the unadjusted HR has a lower CI of 1.00 – suggesting no association, the authors should not overstate an association of unadjusted results.

Thank you for this point – we have removed this sentence.

7. It is difficult to discern what is prenatal exposure vs early childhood exposure in this population. The authors have mentioned this in the limitations. However, I am wondering whether they have data on how many stayed within the exposure area post birth and during the period of water contamination?

Thank you for this insightful question. Most subjects in the very high exposure group lived in an address receiving contaminated water through at least age three. When the contamination was identified and shut off at the end of 2013, the oldest subjects in our cohort had reached age 8. We have expanded on this in the discussion:

“Furthermore, the exposure assessment is based on maternal exposure prior to delivery and doesn’t capture ongoing exposure in childhood. Many children with high or very high prenatal exposure continued living in the contaminated area through 2013; for example, at age three, more than half (57.2%) of the children in the very high prenatal exposure group still resided at an address receiving contaminated water. Therefore, we cannot conclude that the observed effects in this study are due exclusively to prenatal exposure, and further research is needed to clarify which developmental periods are most sensitive to high PFAS exposure.” (Line 472-482).

8. Clarification of the outcome ascertainment around wheeze is needed, it’s unclear why J45 is included in this outcome and may explain the low sensitivity.

Thank you for this helpful comment. The inclusion of J45 (asthma) in the wheeze outcome was intentional, as wheezing in early childhood can represent both early asthma presentations and transient wheezing illnesses. The wheeze outcome was therefore defined to be broader and more inclusive, capturing both asthma and other lower respiratory diagnoses (J20-J22) as well as relevant medications. We have edited the Methods section to explain this rationale more clearly:

“Because asthma and wheeze can be difficult to distinguish in children under three, we created a broader outcome to capture early-life wheeze between ages 0-36 months. This outcome included diagnosis codes for asthma (J45) and acute lower respiratory infections (J20-22) and/or at least one dispensation of asthma- and wheeze-related medication, to encompass both early asthma presentations and transient wheezing illnesses.” (Line 180-186)

While the reviewer is correct that wheeze has lower specificity and sensitivity than the other two outcomes, the low sensitivity is due to cases of acute infections in the lower airways (J20-J22) that are diagnosed in primary care but not in the National Patient Registry. This limitation is included in the discussion section.

## Minor comments

9. The use of sharp null hypothesis should be explained; this will not be a concept familiar to most readers.

Thanks for this helpful suggestion. We have added the following description:

“We then tested the Fisher sharp null hypothesis which states We tested the sharp null hypotheses that each child’s outcome would have been identical under both exposure levels (formally, $H_{0}: Y_{i}\left( W_{i}= 0 \right)= Y_{i}\left( W_{i}= 1 \right)$ for all children i) using the difference in cumulative incidence as our test statistic [61,62]. Under this null hypothesis, treatment assignment can be viewed as hypothetically randomized within each matched set of four children, allowing us to approximate one-sided Fisher’s exact p-values for each outcome with 100,000 exposure permutations [60]difference in outcome-specific incidence between background- and very high-prenatal PFAS exposure, where exposure assignment was assumed to be randomized within each set of four matched children [63,64].” (Line 289-298)

10. The validation of the exposure is a significant strength of the study and analysis appropriate. Given the samples were collected from 2014 – 2016, were there differences between those collected remote from exposure which may have influenced levels?

Thank you for raising this point. The samples collected in 2014-2016, after the contaminated water supply had been identified and replaced. We have clarified this in our discussion:

“Although PFAS concentrations could not be measured directly in the study participants, we validated our exposure classification in a separate cohort of women of childbearing age. Samples in the validation cohort were collected after the contaminated drinking water supply had been identified and replaced (2014-2016), which likely led to some underestimation of true exposure levels in our study population.” (Line 421-425)

Because serum samples were collected shortly after exposure ended, some temporal decline in PFAS concentrations in the exposed population is expected. Levels of PFOS and PFOA in the Swedish unexposed population have also decreased over time (Norén et al., 2021). However, the exposure contrast in Ronneby was extremely large, so these temporal changes would not meaningfully affect the separation between exposure categories. Participants in the validation cohort were classified based on their residential histories during the exposure period, ensuring that any migration after the exposure event did not bias the results.

11. The higher level of exposure in the present study should be a greater focus in the discussion of previous studies as this has likely contributed to the differences.

Thank you for this important point. We have rewritten the discussion section to emphasize this point, starting with Line 415:

“Our study differs from previous epidemiological investigations by including a subset of participants with high exposure to AFFF-associated PFAS, yielding a much wider range of prenatal PFAS levels than typically observed in background-exposed populations.”

12. Discussion of the applicability of the present study to the general population, given such very high levels are likely only rarely seen.

Thank you for this important point. We agree that the results from the very-high exposure group are not necessarily applicable to populations exposed only to background PFAS levels. We have clarified this in the discussion, and expanded our concluding paragraph to contextualize the relevance of our findings:

“In this study, we observed an elevated risk of asthma among children with very high prenatal PFAS exposure from AFFF-contaminated drinking water. These findings may not generalize to populations with lower exposures or different PFAS mixtures, as exposure-response relationships remain uncertain and individual compounds vary in toxicity. However, AFFF-related PFAS contamination is a major source of high environmental exposure globally [18,109], and evidence from Ronneby offers important insights into the potential health effects of such contamination in affected communities. Replication in other highly exposed populations is needed to confirm these results, but they point to a substantial and previously unrecognized public health consequence of AFFF-related PFAS contamination.” (Line 520-533)

13. The use of maternal address as a proxy should be noted as a limitation.

Thank you, we agree that this is an important limitation for this study. We discuss this in detail, starting with Line 465:

“A final study limitation is its address-based categorical exposure assessment. This approach may induce non-differential exposure error...”

14. Parental asthma will not capture childhood asthma among parents this should be noted as a limitation.

We have added this point to the limitations section:

“Our study could not account for parental history of childhood asthma, but we adjusted for prevalent parental asthma during the study period” (Line 442-444)

15. Supplemental figures should include point estimates and 95% CI so they can be compared with main analysis.

Thank you for this suggestion. We have added S6 Table and S7 Table to the Supplemental Material with the relevant model information from S12 Figure and S13 Figure, including point estimates and confidence intervals.

# Reviewer 2

16. Line 84: Please consider using a flow chart to display the participant selection process.

Thank you for this suggestion. We did consider including a flow chart, as they are standard in most studies. However, this is a registry-based study that includes the entire population of Blekinge born between 2006-2013, who were followed from birth until outcome incidence, death, maximum age, or censoring. The only exclusion criteria was complete covariate information, as described on Line XXX:

“Of the 12,585 children in our cohort born in Blekinge between 2006 and 2013, 11,488 (92%) had complete covariate information and were included in our final study population.” (Line 316-317)

For this reason, we considered the description of the cohort in the paper and in Table S1 to be sufficient.

17. Line 130: Given that the study period spans 2006 to 2013 while the validation study for exposure assessment was conducted from 2014 to 2016, please consider performing a sensitivity analysis that restricts the study period to the final years or latter half, where the validation study findings are more likely to be applicable.

Thank you for this suggestion. We appreciate that the results from the validation study (2014-2016) may not fully reflect exposure levels in the population from 2006-2013. We have made this more explicit in our discussion:

“Although PFAS concentrations could not be measured directly in the study participants, we validated our exposure classification in a separate cohort of women of childbearing age. Samples in the validation cohort were collected after the contaminated drinking water supply had been identified and replaced (2014-2016), which likely led to some underestimation of true exposure levels in our study population.” (Line 421-425)

Based on your comment, we have also completed a sensitivity analysis limiting our study population to subjects born 2010-2013. Given the limited space, we have decided against incorporating this sensitivity analysis into our final manuscript. However, we have provided it here for your reference.

Table 1: Children (N) by exposure category in the entire cohort and in the cohort limited to children born between 2010-2013

| Prenatal Exposure Group | N | |
| --- | --- | --- |
|  | Entire cohort | Limited 2010-2013 |
| Background | 9224 | 4472 |
| Intermediate | 1591 | 757 |
| High | 479 | 234 |
| Very High | 194 | 100 |

Table 2: Hazard Ratios for models estimated from the entire cohort compared to a sensitivity cohort of children born between 2010-2013.

| Prenatal Exposure Group | Events | | Adjusted Hazard Ratio (95% CI) | |
| --- | --- | --- | --- | --- |
|  | Entire cohort | Limited 2010-2013 | Entire cohort | Limited 2010-2013 |
| **Wheeze** |  |  |  |  |
| Background | 1661 | 798 (18%) | - |  |
| Intermediate | 284 | 138 (18%) | 0.99 (0.88, 1.13) | 1.03 (0.86, 1.23) |
| High | 103 | 57 (24%) | 1.09 (0.89, 1.33) | 1.29 (0.98, 1.69) |
| Very High | 37 | 22 (22%) | 0.97 (0.70, 1.35) | 1.12 (0.73, 1.72) |
| **Asthma** |  |  |  |  |
| Background | 1596 | 719 (16%) | - |  |
| Intermediate | 261 (16%) | 123 (16%) | 0.95 (0.83, 1.08) | 1.02 (0.84, 1.23) |
| High | 89 (19%) | 39 (17%) | 0.96 (0.78, 1.19) | 0.92 (0.67, 1.28) |
| Very High | 50 (26%) | 26 26%) | 1.44 (1.09, 1.91) | 1.56 (1.05, 2.31) |
| Asthma (3+) |  |  |  |  |
| Background | 1170 (13%) | 515 (12%) | - | - |
| Intermediate | 186 (12%) | 89 (12%) | 0.93 (0.79, 1.08) | 1.04 (0.83, 1.30) |
| High | 63 (13%) | 28 (12%) | 0.94 (0.73, 1.22) | 0.93 (0.63, 1.36) |
| Very High | 40 (21%) | 20 (20%) | 1.59 (1.16, 2.18) | 1.72 (1.09, 2.69) |

18. Line 138: Please provide a brief description of the Swedish medical system, although this point is addressed in the study limitations section.

Thank you for this suggestion. We have added the following description:

“Swedish healthcare is publicly funded and free of charge for all children up to the age of 18 years [33].”(Line 164)

19. Line 184: The authors appear to use two different methods to indicate asthma presence (true/false or yes/no). Please clarify this inconsistency.

Thank you for your attention to detail and for catching this inconsistency – we have changed the text to yes/no throughout.

20. Line 202: Please provide details of the procedures used to test the proportional hazard assumptions.

Thank you for this suggestion. We have added this information to the manuscript.

*Methods*: “The proportional hazards assumption was evaluated for each covariate using scaled Schoenfeld residuals regressed against a Kaplan–Meier transformation of time [51]. We formally assessed the proportional hazards assumption for our categorical exposure variable by jointly testing the time-by-exposure interaction terms using robust Wald tests, which account for the cluster-robust covariance estimates from the fitted Cox models.” (Line 238-243)

*Results*: “For all outcomes, robust Wald tests for the exposure-by-time interaction terms indicated no evidence of non-proportional hazards by exposure category (wheeze: p=0.71; asthma: p = 0.14; asthma (3+): p=0.29).” (Line 327-329)

21. Line 435: The authors stated that children in the high exposure group continued to experience postnatal exposure. This assertion appears inaccurate for children born during the 2010s. Did area residents continue consuming contaminated water subsequent to 2013?

Thank you for raising this important point. You are right - residents did not continue to consume contaminated water after 2013. We have changed the discussion to be more specific:

“Furthermore, the exposure assessment is based on maternal exposure prior to delivery and doesn’t capture ongoing exposure in childhood. Many children with high or very high prenatal exposure continued living in the contaminated area through 2013; for example, at age three, more than half (57.2%) of the children in the very high prenatal exposure group still resided at an address receiving contaminated water.” (Line 472-479).

# References

Bind, M.-A.C., Rubin, D.B., 2019. Bridging observational studies and randomized experiments by embedding the former in the latter. Stat Methods Med Res 28, 1958–1978. https://doi.org/10.1177/0962280217740609

Norén, E., Lindh, C., Glynn, A., Rylander, L., Pineda, D., Nielsen, C., 2021. Temporal trends, 2000–2017, of perfluoroalkyl acid (PFAA) concentrations in serum of Swedish adolescents. Environment International 155, 106716. https://doi.org/10.1016/j.envint.2021.106716

# Editorial Comments

## General editorial requests:

1. We ask every co-author listed on the manuscript to fill in a contributing author statement, making sure to declare all competing interests. If any of the co-authors have not filled in the statement, we will remind them to do so when the paper is revised. If all statements are not completed in a timely fashion this could hold up the re-review process. If new competing interests are declared later in the revision process, this may also hold up the submission. Should there be a problem getting one of your co-authors to fill in a statement we will be in contact. Please do not add or remove authors without first discussing this with the handling editor. You can see our competing interests policy here: http://journals.plos.org/plosmedicine/s/competing-interests.

All co-authors agree to complete a contributing author statement, if they have not yet.

2. Please upload any figures associated with your paper as individual TIF or EPS files with 300dpi resolution at resubmission; please read our figure guidelines for more information on our requirements: http://journals.plos.org/plosmedicine/s/figures. While revising your submission, we strongly recommend that you use PLOS's NAAS tool (https://ngplosjournals.pagemajik.ai/artanalysis) to test your figure files. NAAS can convert your figure files to the TIFF file type and meet basic requirements (such as print size, resolution), or provide you with a report on issues that do not meet our requirements and that NAAS cannot fix.

Figures have been uploaded as EPS files and meet the journal requirements.

3. Please ensure that the paper adheres to the PLOS Data Availability Policy (see http://journals.plos.org/plosmedicine/s/data-availability), which requires that all data underlying the study's findings be provided in a repository or as Supporting Information. For data residing with a third party, authors are required to provide instructions with contact information (web or email address) for obtaining the data. Please note that a study author cannot be the contact person for the data. PLOS journals do not allow statements supported by "data not shown" or "unpublished results." For such statements, authors must provide supporting data or cite public sources that include it.
4. We expect all researchers with submissions to PLOS in which author-generated code underpins the findings in the manuscript to make all author-generated code available without restrictions upon publication of the work. In cases where code is central to the manuscript, we may require the code to be made available as a condition of publication. Authors are responsible for ensuring that the code is reusable and well documented. Please make any custom code available, either as part of your data deposition or as a supplementary file. Please add a sentence to your data availability statement regarding any code used in the study, e.g. "The code used in the analysis is available from Github [URL] and archived in Zenodo [DOI link]" Please review our guidelines at https://journals.plos.org/plosmedicine/s/materials-software-and-code-sharing and ensure that your code is shared in a way that follows best practice and facilitates reproducibility and reuse. Because Github depositions can be readily changed or deleted, we encourage you to make a permanent DOI'd copy (e.g. in Zenodo) and provide the URL.

Our submission follows the PLOS Data Availability Policy and includes all author-generated code used in the analyses. This is specified in our new Data Availability Statement:

“Data cannot be shared publicly because it contains sensitive personal information. Researchers can apply for data access at each of the relevant Swedish registry holders (Region Blekinge, Statistics Sweden, and the Swedish National Board of Health and Welfare) for research projects that have been approved by the Swedish Ethical Review Authority. The analysis code and results are openly available in a published GitHub repository (https://github.com/ajblomberg/PFAS-and-Childhood-Asthma) that has been permanently archived on Zenodo with a DOI (https://doi.org/10.5281/zenodo.17931667).”

## Formatting – general

5. Abstract: Please structure your abstract using the PLOS Medicine headings (Background, Methods and Findings, Conclusions). Please combine the Methods and Findings sections into one section.

Our abstract meets this structure.

6. At this stage, we ask that you include a short, non-technical Author Summary of your research to make findings accessible to a wide audience that includes both scientists and non-scientists. The Author Summary should immediately follow the Abstract in your revised manuscript. This text is subject to editorial change and should be distinct from the scientific abstract. Ideally each sub-heading should contain 2-3 single sentence, concise bullet points containing the most salient points from your study. In the final bullet point of 'What Do These Findings Mean?', please include the main limitations of the study in non-technical language. Please see our author guidelines for more information: https://journals.plos.org/plosmedicine/s/revising-your-manuscript#loc-author-summary.

This has been added.

7. Please express the main results with 95% CIs as well as p values. When reporting p values please report as p<0.001 and where higher as the exact p value p=0.002, for example. Throughout, suggest reporting statistical information as follows to improve clarity for the reader "22% (95% CI [13%,28%]; p</=)". Please be sure to define all numerical values at first use.

This formatting has been implemented throughout.

8. Please include page numbers and line numbers in the manuscript file. Use continuous line numbers (do not restart the numbering on each page).

This has been implemented.

9. Please cite the reference numbers in square brackets. Citations should precede punctuation.

This is implemented throughout.

## Figures and Tables

10. Please provide titles and legends for all figures and tables (including those in Supporting Information files).

Titles and legends for figures and tables have been included in the submission.

11. Please define all abbreviations used in each figure/table (including those in Supporting Information files).

All abbreviations have been defined.

12. Please consider avoiding the use of red and green in order to make your figure more accessible to those with color blindness.

Figure colors were selected to be colorblind safe and print-friendly (ColorBrewer - Qualitative Dark2)

## Supplementary Material

Please note that supplementary material will be posted as supplied by the authors. Therefore, please amend it according to the relevant comments outlined here.

13. Please cite your Supporting Information as outlined here: https://journals.plos.org/plosmedicine/s/supporting-information

This has been implemented as recommended.

## References

14. PLOS uses the numbered citation (citation-sequence) method and first six authors, et al.

We have followed the PLOS citation style.

15. Please ensure that journal name abbreviations match those found in the National Center for Biotechnology Information (NCBI) databases (http://www.ncbi.nlm.nih.gov/nlmcatalog/journals), and are appropriately formatted and capitalised.

Thank you, this has been completed.

16. Where website addresses are cited, please include the complete URL and specify the date of access (e.g. [accessed: 12/06/2023]).

This has been completed.

17. Please also see https://journals.plos.org/plosmedicine/s/submission-guidelines#loc-references for further details on reference formatting.

To the best of our knowledge, we have met all guidelines on reference formatting.

## Observational Studies

1. Abstract: Please include the study design, population and setting, number of participants, years during which the study took place (enrollment and follow up), length of follow up, and main outcome measures.

Thank you – this information is now included in the abstract.

2. Please ensure that the study is reported according to the STROBE (or appropriate STOBE extension) guideline (available from: https://www.equator-network.org/reporting-guidelines/strobe) and include the completed STROBE (or STROBE extension) checklist as Supporting Information. Please add the following statement, or similar, to the Methods: "This study is reported as per the Strengthening the Reporting of Observational Studies in Epidemiology (STROBE) guideline (S1 Checklist)." When completing the checklist, please use section and paragraph numbers, rather than page numbers. If more appropriate, please ensure that the study is reported according to the RECORD guideline (available from [https://www.record-statement.org)]https://www.record-statement.org) and include the completed checklist as Supporting Information. Please add the following statement, or similar, to the Methods: "This study is reported as per the Reporting of Studies Conducted using Observational Routinely-Collected Data (RECORD) guideline (S1 Checklist)." When completing the checklist, please use section and paragraph numbers, rather than page numbers.

The STROBE checklist is included as S1 Strobe Checklist.

3. For all observational studies, in the manuscript text, please indicate: (1) the specific hypotheses you intended to test, (2) the analytical methods by which you planned to test them, (3) the analyses you actually performed, and (4) when reported analyses differ from those that were planned, transparent explanations for differences that affect the reliability of the study's results. If a reported analysis was performed based on an interesting but unanticipated pattern in the data, please be clear that the analysis was data driven.

This information is included in the study.

4. Please state in the Methods section whether the study had a prospective protocol or analysis plan. If a prospective analysis plan (from your funding proposal, IRB or other ethics committee submission, study protocol, or other planning document written before analyzing the data) was used in designing the study, please include the relevant document(s) with your revised manuscript as a Supporting Information file to be published alongside your study and cite it in the Methods section. A legend for this file should be included at the end of your manuscript. If no such document exists, please make sure that the Methods section transparently describes when analyses were planned, and when/why any data-driven changes to analyses took place. Changes in the analysis, including those made in response to peer review comments, should be identified as such in the Methods section of the paper, with rationale.

This has been completed.
