# Supplementary material for: Prenatal exposure to per- and polyfluoroalkyl substances (PFAS) and incidence of asthma and wheeze in childhood: A register-based cohort study in Ronneby, Sweden
Source: PLoS Med. 2026 Apr 9;23(4):e1004659. doi: 10.1371/journal.pmed.1004659 (PMC13065015; doi:10.1371/journal.pmed.1004659)
Supplement: Attachment — Submitted filename: Reviewer-and-Editorial-Responses-3-1-1.docx [file pmed.1004659.s019.docx]

Response to Editor Comments: PMEDICINE-D-25-01709-R2

Dear Editors and reviewers:

Thank you for the opportunity to revise our manuscript for publication. We would also like to extend our thanks to Reviewer 3 for their thoughtful and detailed consideration of the manuscript. Below are point-by-point responses to the editorial and reviewer comments. All line numbers refer to our clean manuscript that does not include tracked changes.

# General Editorial Requests

1. Please confirm that your title complies with PLOS Medicine's style. Your title must be nondeclarative and not a question. It should begin with main concept if possible. "Effect of" should be used only if causality can be inferred, i.e., for an RCT. Please place the study design ("A randomized controlled trial," "A retrospective study," "A modelling study," etc.) in the subtitle (ie, after a colon).

We can confirm that our title, “Prenatal exposure to perfluoroalkyl substances (PFAS) and incidence of asthma and wheeze in childhood: A cohort study in Ronneby, Sweden” complies with PLOS Medicine’s style guidelines.

2. Please confirm that your abstract complies with our requirements, including format (three sections: Background, Methods and Findings, and Conclusions) and providing all the information relevant to this study type.

Our abstract complies with PLOS Medicine requirements. Changes to the abstract are noted in Question 9 and 10.

3. Please ensure that the Introduction ends with a clear description of the study question or hypothesis.

Our introduction section concludes with a clear statement of the study hypothesis: “We hypothesized that children born to mothers who lived in the high-exposed area of Ronneby would have increased incidence of wheeze and asthma.”

4. Please ensure that all abbreviations are defined at first use throughout the text.

This is the case.

5. Please confirm that all numbers presented in the abstract are present and identical to numbers presented in the main manuscript text.

All numbers in the abstract match numbers from the main manuscript text.

6. Please review your text for claims of novelty or primacy (e.g. 'for the first time') and remove this language. In addition, please check that any use of statistical terms (such as trend or significant) are supported by the data, and if not please remove them.

Thank you for this comment. One claim of novelty in the discussion has been removed.

7. Please remove the 'conclusions' subheading from the discussion. Please also remove any other subheadings from the discussion.

All subheadings in the discussion have been removed.

8. Statistical reporting: Please revise throughout the manuscript, including tables and figures.

- Please report statistical information as follows to improve clarity for the reader (95% CI [13,28]; p</=).
- Please separate upper and lower bounds with commas instead of hyphens as the latter can be confused with reporting of negative values.
- Please repeat statistical definitions (HR, CI etc.) for each set of parentheses.

The one instance of a hyphen being used instead of a comma in an upper and lower bound (in the abstract) has been corrected. We also confirm that the statistical definitions have also been repeated for all reported effect estimates.

9. In the last sentence of the Abstract Methods and Findings section, please describe the main limitation(s) of the study's methodology.

Thank you, this has been added:

Line 43: “Study limitations include its reliance on an address-based categorical proxy for prenatal PFAS exposure, which induces non-differential exposure misclassification and limits separation of prenatal and early-childhood exposure effects.”

10. In the abstract, please include the important dependent variables that are adjusted for in the analyses.

Thank you, this has been added:

Line 31: “We identified incident cases of wheeze and asthma from administrative health records and estimated hazard ratios (HRs) using Cox proportional hazards models adjusted for individual-level confounders, including maternal smoking in early pregnancy, maternal age at delivery, parity, child sex, parental asthma, and socioeconomic factors.”

11. Please replace "subject" with participant, patient, individual, or person.

We have made this change throughout the manuscript.

12. The funding statement should include: specific grant numbers, initials of authors who received each award, URLs to sponsors’ websites. Also, please state whether any sponsors or funders (other than the named authors) played any role in study design, data collection and analysis, the decision to publish, or preparation of the manuscript. If they had no role in the research, include this sentence: “The funders had no role in study design, data collection and analysis, decision to publish, or preparation of the manuscript.

The funding statement matches these guidelines. URLs to the funder websites have been added as hyperlinks.

13. All authors must declare their relevant competing interests per the PLOS policy, which can be seen here: [https://journals.plos.org/plosmedicine/s/competing-interests](https://journals.plos.org/plosmedicine/s/competing-interests "https://journals.plos.org/plosmedicine/s/competing-interests") For authors with ties to industry, please indicate whether any of the interests has a financial stake in the results of the current study.

The authors have declared that no competing interests exist.

14. PLOS Medicine requires that the de-identified data underlying the specific results in a published article be made available, without restrictions on access, in a public repository or as Supporting Information at the time of article publication, provided it is legal and ethical to do so. Please see the policy at <http://journals.plos.org/plosmedicine/s/data-availability> and FAQs at <http://journals.plos.org/plosmedicine/s/data-availability#loc-faqs-for-data-policy>. The Data Availability Statement (DAS) requires revision. For each data source used in your study:

a) If the data are freely or publicly available, note this and state the location of the data: within the paper, in Supporting Information files, or in a public repository (include the DOI or accession number).

b) If the data are owned by a third party but freely available upon request, please note this and state the owner of the data set and contact information for data requests (web or email address). Note that a study author cannot be the contact person for the data.

c) If the data are not freely available, please describe briefly the ethical, legal, or contractual restriction that prevents you from sharing it. Please also include an appropriate contact (web or email address) for inquiries (again, this cannot be a study author).

We have rewritten the DAS to comply with the guidelines provided above. Please let us know if further revisions are needed.

Line 862: Data cannot be shared publicly because they contain sensitive personal information and are subject to legal and ethical restrictions under Swedish law. Access to the data is restricted to research projects approved by the Swedish Ethical Review Authority and the relevant registry holders; for some registers, access is limited to researchers affiliated with Swedish institutions. Applications for data access must be submitted directly to the respective registry owners. Further details on data access procedures are available at the websites provided below.

- Swedish National Board of Health and Welfare (https://bestalladata.socialstyrelsen.se/data-for-forskning/): National Patient Register, National Prescribed Drug Register, and National Medical Birth Register.
- Statistics Sweden (https://www.scb.se/vara-tjanster/bestall-data-och-statistik/mikrodata/): Total Population Register and the Longitudinal Integrated Database for Health Insurance and Labor Market Studies (LISA).
- Region Blekinge (https://regionblekinge.se/halsa-och-vard/forskning-och-utveckling.html): Blekinge Healthcare Register.

The analysis code and results are openly available in a published GitHub repository (https://github.com/ajblomberg/PFAS-and-Childhood-Asthma) that has been permanently archived on Zenodo with a DOI (<https://doi.org/10.5281/zenodo.17931667>).

15. Please specify the reason informed consent was not required. Please ensure that the research complies with the PLOS policy in full:

We have updated our text to specify that informed consent was not required for the primary analyses and why:

Line 160: “The Swedish Ethical Review Authority approved the study (number 2021-04872) and waived the requirement for individual informed consent because the research used existing registry data and could not practicably be conducted if consent were required.”

We have also added information about informed consent and ethical approval for the Ronneby Biomarker Cohort:

Line 191: ***“***Written informed consent was received from all participants in the Ronneby Biomarker Cohort, which was approved by the Regional Ethical Review Board in Lund, Sweden (number 2014/4).***”***

We confirm that our study complies with the PLOS policy.

## Figures

16. Please define all elements of box plots in the figure caption - center line, box limits and whiskers.

This has been added to the S9 Figure caption:

“PFAS concentrations (ng/mL) in a subset of the Ronneby Biomarker Cohort (female subjects aged 21-40 years, N = 209), stratified by categorical exposure groups. The box plots show the median (center line), interquartile range (box), and whiskers extending to 1.5 times the interquartile range; individual observations are shown in blue.”

17. Please provide titles and legends for all figures and tables (including those in Supporting Information files). Please define all acronyms used in each figure or table in its corresponding legend.

We confirm that all figures and tables include titles and legends. All abbreviations and shorthand terms have been defined. In addition, we have replaced abbreviations where appropriate (e.g., IQR to “interquartile range”, SEK to “Swedish krona” and CI to “Confidence Interval”). In S11 Figure, Q2, Q3, and Q4 are now defined, and in S14 Figure, the shorthand used in the legend is now clarified.

18. Please ensure that where relevant figures include 95% CIs.

All relevant figures include 95% CIs.

## OBSERVATIONAL, COHORT, CROSS-SECTIONAL, AND CASE CONTROL STUDIES

19. Please ensure that the study is reported according to the STROBE guideline, and include the completed STROBE checklist as Supporting Information.  Please add the following statement, or similar, to the Methods: ""This study is reported as per the Strengthening the Reporting of Observational Studies in Epidemiology (STROBE) guideline (S1 Checklist).

The study is reported according to both the STROBE and RECORD guidelines. The RECORD guidelines have been included as Supporting Information (see question 24).

20. When completing the checklist, please use section and paragraph numbers, rather than page numbers.

The RECORD checklist is completed with section and paragraph numbers, not page numbers.

21. Did your study have a prospective protocol or analysis plan? Please state this (either way) early in the Methods section.

a) If a prospective analysis plan (from your funding proposal, IRB or other ethics committee submission, study protocol, or other planning document written before analyzing the data) was used in designing the study, please include the relevant prospectively written document with your revised manuscript as a Supporting Information file to be published alongside your study, and cite it in the Methods section. A legend for this file should be included at the end of your manuscript.
b) If no such document exists, please make sure that the Methods section transparently describes when analyses were planned, and when/why any data-driven changes to analyses took place.

In either case, changes in the analysis-- including those made in response to peer review comments-- should be identified as such in the Methods section of the paper, with rationale.

Thank you for this important point. In the beginning of the Methods section we now specify that our study did not have a prospective protocol:

Line 162: “The study was conducted within a broader ethically approved research program; however, no study-specific prospective protocol or statistical analysis plan was developed for the present investigation.”

We have also clarified which aspects of the statistical analysis were data-informed (exposure categorization), suggested by peer review (time-by-exposure testing of proportional hazards assumptions), or introduced as a secondary analysis (Rubin Causal Model).

Line 179: “Although earlier Ronneby registry studies used a three-level exposure categorization [32,33], we applied a four-level categorization supported by exposure validation analyses indicating stepwise increases in measured PFAS concentrations across the four groups.”

Line 272: “We formally assessed the proportional hazards assumption for our categorical exposure variable by jointly testing the time-by-exposure interaction terms using robust Wald tests, which account for the cluster-robust covariance estimates from the fitted Cox models; this assessment was added in response to peer review.”

Line 290: “Following the primary survival analyses, we conducted a secondary analysis using a Rubin Causal Model potential outcomes framework.”

22. Your study is observational and therefore causality cannot be inferred. Please remove language that implies causality and refer to associations instead.

We have removed all causal language from the manuscript, including in the Abstract, Author Summary, and last paragraph of the Discussion.

23. For all observational studies, in the manuscript text, please indicate: (1) the specific hypotheses you intended to test, (2) the analytical methods by which you planned to test them, (3) the analyses you actually performed, and (4) when reported analyses differ from those that were planned, transparent explanations for differences that affect the reliability of the study's results. If a reported analysis was performed based on an interesting but unanticipated pattern in the data, please be clear that the analysis was data-driven.

Thank you for this clarification.

(1) The specific study hypothesis is stated at the end of the Introduction: “We hypothesized that children born to mothers who lived in the high-exposed area of Ronneby would have increased incidence of wheeze and asthma.”

(2) The analytical methods planned to test this hypothesis are described in the Statistical Analysis section, and include Kaplan–Meier visualizations and Cox proportional hazards models adjusted for prespecified covariates identified via a directed acyclic graph.

(3) The analyses that were performed are fully detailed in the Methods section, including exposure validation and outcome validation, survival modeling, multiple imputation, and the secondary Rubin Causal Model analysis.

(4) Where analyses differed from those initially planned, these differences are now explicitly identified in the Methods section. Specifically, we clarify that the four-level exposure categorization was informed by exposure validation results; that formal time-by-exposure testing of the proportional hazards assumption was added in response to peer review; and that the Rubin Causal Model analysis was conducted as a secondary analysis following completion of the primary survival models. No analyses were undertaken in response to unanticipated patterns in the outcome data.

24. If more appropriate than STROBE, please ensure that the study is reported according to the RECORD guideline (available from [https://www.record-statement.org)]https://www.record-statement.org) and include the completed checklist as Supporting Information. Please add the following statement, or similar, to the Methods: "This study is reported as per the Reporting of Studies Conducted using Observational Routinely-Collected Data (RECORD) guideline (S1 Checklist)." When completing the checklist, please use section and paragraph numbers, rather than page numbers

Thank you for this suggestion. Given the relevance of the RECORD statement to our study, we have completed the checklist and included it as S1 Record Checklist. This is now stated in the Methods section:

Line 314: “We reported our study following the Reporting of Studies Conducted using Observational Routinely-Collected Data (RECORD) Statement (S1 RECORD Checklist)”

# Comments from Reviewers

## Reviewer 3

25. Abstract: One minor suggestion: in the "Methods and Findings" summary, consider specifying that the increased asthma risk was observed specifically in the very high exposure group, as this is a crucial nuance.

Thank you for this suggestion. We now specify this in the abstract “Methods and Findings” section:

Line 39: “Very high prenatal PFAS exposure was associated with incidence of asthma (HR: 1.44, 95% CI [1.08, 1.92]), whereas no associations were observed in the high or intermediate exposure groups or for wheeze.”

26. Exposure Assessment: In the main text or supplement, consider adding a brief discussion of the potential implications of using pre-delivery maternal address as a proxy for fetal exposure, acknowledging it as a reasonable but imperfect measure.

Thank you for this point. We have added a brief description of the proxy measure’s limitations to the exposure assessment section. A detailed discussion of its implications is included in the Discussion section.

Line 176: “As a proxy for fetal PFAS exposure, this categorization assumes that maternal residential history reflects drinking water exposure before and during pregnancy, and does not account for individual differences in water consumption or temporal changes in PFAS concentrations.”

27. Results: When presenting the sex-stratified results in the text or supplement, a brief interpretation of the marginal interaction for wheeze (p=0.08) and the direction of effects could be helpful, even if not statistically definitive.

This is a helpful clarification to raise - thank you. The manuscript now includes a more direct interpretation of the differences in PFAS-wheeze association by sex:

Line 343: “In models including sex-by-exposure interaction terms, there was suggestive evidence of effect modification for wheeze (p=0.08), but not for asthma (p=0.33) or asthma (3+) (p=0.52). Sex-stratified models for wheeze indicated that very high PFAS exposure, compared to background exposure, was associated with a lower hazard of wheeze in girls (HR: 0.50, 95% CI [0.24, 1.04]) and a higher hazard in boys (HR: 1.23, 95% CI [0.84, 1.79]). However, these sex-specific estimates were imprecise and included the null value (S7 Table and S13 Figure).

28. Discussion - Strengths: You may consider adding the large sample size and the use of time-to-event analysis as explicit strengths.

Thank you for the suggestion. We have rewritten the strengths section to clearly address the large sample size and time-to-event analysis:

Line 455: “This study has several additional strengths. The large population-based cohort (N = 11,488) included detailed longitudinal information on time-to-event and censoring, permitting survival analysis of asthma incidence and efficient use of follow-up time [108,109].”

29. Discussion - Limitations: The discussion of exposure misclassification is excellent and appropriately nuanced (differentiating error structures). The point about ongoing childhood exposure for many children is critical and is well-handled. You might briefly reiterate here that this precludes attributing effects solely to the prenatal period.

In response to this suggestion, we have revised the paragraph on exposure misclassification to clearly conclude that associations cannot be attributed solely to the prenatal period:

Line 441: “Furthermore, the exposure assessment is based on maternal exposure prior to delivery and does not capture ongoing exposure in childhood. Many children with high or very high prenatal exposure continued living in the contaminated area through 2013; for example, at age three, more than half (57%) of the children in the very high prenatal exposure group still resided at an address receiving contaminated water. Given the likelihood of continued postnatal exposure, we cannot attribute the observed associations solely to prenatal exposure, and further research is needed to clarify which developmental periods are most sensitive to high PFAS exposure.”

30. Conclusions: The conclusions are balanced and justified. Consider softening the statement "may not generalize to populations with lower exposures" to "likely have limited generalizability to populations with only background-level exposures," as this aligns with your threshold-effect hypothesis.

Thank you for raising this subtle point: we have made this change in the manuscript.
