# Supplementary material for: Prenatal exposure to per- and polyfluoroalkyl substances (PFAS) and incidence of asthma and wheeze in childhood: A register-based cohort study in Ronneby, Sweden
Source: PLoS Med. 2026 Apr 9;23(4):e1004659. doi: 10.1371/journal.pmed.1004659 (PMC13065015; doi:10.1371/journal.pmed.1004659)
Supplement: Attachment — Submitted filename: Reviewer-and-Editorial-Responses-4-1-1.docx [file pmed.1004659.s020.docx]

Response to Editor Comments: PMEDICINE-D-25-01709-R3

Dear Editors:

Thank you for the opportunity to continue to improve our manuscript for publication. Below are point-by-point responses to the editorial comments. All line numbers refer to our clean manuscript that does not include tracked changes.

# General Editorial Requests

1. Please ensure the Abstract is proofread, no sentence starts with a number and the acronym AFFF is defined on first use.

Thank you, we have edited the abstract and made these suggested changes.

2. Please add the limitations of the study as a last point in the author summary.

This has been added.

3. The funding statement should include URLs to the sponsors’ websites rather than hyperlinks.

We have made this change.

4. Please clarify if the data was anonymized and whether this was the reason informed consent was not required.

Thank you for this request. We have clarified in the Methods section that data linkage was performed by Statistics Sweden and that the dataset was de-identified prior to delivery to the research team. Pseudonymization is required for all register-based research in Sweden.

The requirement for individual informed consent was waived by the Swedish Ethical Review Authority (2021-04872). As stated in the manuscript, the waiver was granted because the study was based on existing nationwide registry data and could not practicably be conducted if individual consent were required. The waiver was therefore based on formal ethical review and the nature of the study design, rather than solely on the pseudonymization of the data.

5. Please remove any mention of suggestive evidence if not statistically significant and simply describe the results. We appreciate this is not in line with Reviewer 4 but it is a journal policy requirement.

We have revised the paragraph to remove interpretive language regarding non-statistically significant findings and to describe the results directly. The revised text now reads:

“In models including sex-by-exposure interaction terms, the interaction p-value was lower for wheeze (p=0.08) than for asthma (p=0.33) or asthma (3+) (p=0.52). In sex-stratified models for wheeze, the hazard ratio comparing very high with background exposure differed in direction between girls (HR: 0.50, 95% CI [0.24, 1.04]) and boys (HR: 1.23, 95% CI [0.84, 1.79]) (S7 Table and S13 Figure)”

6. Since PFAS after the prenatal period cannot be ruled out, please consider if using “early-life exposure instead” in the title and the text might be more accurate.

We appreciate this thoughtful suggestion. After discussion within the author group, we have retained the term “prenatal” because the exposure variable was specifically designed to capture maternal residential exposure during pregnancy. The classification is conceptually and temporally anchored to the prenatal period, and thus “prenatal exposure” most accurately reflects the exposure definition used in the study.

We agree that postnatal exposure cannot be fully separated from prenatal exposure in this setting. This limitation is explicitly acknowledged in the manuscript, including in the Abstract and Discussion, where we note the potential for correlated early-life exposure and the resulting constraints on causal interpretation.
